# Public perception of chiropractic in the Taiwanese population: a cross-sectional survey

**DOI:** 10.1186/s12998-025-00571-6

**Published:** 2025-03-11

**Authors:** Han-Hao Chang, Katie de Luca, Matthew Fernandez, Ann Quinton

**Affiliations:** 1 School Health, Medical and Applied Sciences, CQUniversity, Brisbane, QLD Australia; 2 School Health, Medical and Applied Sciences, CQUniversity, Sydney, NSW Australia

**Keywords:** Taiwanese, Taiwan, Chiropractic, Perception, Survey

## Abstract

**Background:**

Research on perception of chiropractic is abundant in Western contexts, yet sparse in Asia. This study aims to bridge this gap by examining the perceptions of chiropractic among Taiwanese adults, focusing on demographics, utilisation, beliefs, and understanding.

**Methods:**

An adapted survey with 27 close-ended items was administered to assess Taiwanese adults’ perception of chiropractic. The electronic survey, using Qualtrics, was delivered worldwide via Taiwanese Facebook groups from January 31 to March 31, 2024. Descriptive statistics, including frequencies and cross tabulations, were performed.

**Results:**

A total of 769 individuals were surveyed, with 475 participants providing complete data. Over half of the participants (62%) had never visited a chiropractor, but in those who had visited a chiropractor 78% reported satisfaction. Of 475 participants, 45% considered chiropractic care safe while 34% were unsure. Though almost half (42%) were unclear about what chiropractors do, most participants (67%) expressed interest in learning more. Among the 151 participants with prior experience of chiropractic care, the demographic profile was 54% women, and individuals aged 28 to 37 (44%), and those with an undergraduate degree (52%) were most common.

**Conclusion:**

Overall, our study found a positive perception and high acceptance of chiropractic among the Taiwanese population; however, generalisability may be limited due to the risk of selection bias. An understanding of the chiropractic profession was notably limited. Hence, efforts are needed to enhance awareness of chiropractic accreditation, clinical competencies, and its potential role in public healthcare in Taiwan.

**Supplementary Information:**

The online version contains supplementary material available at 10.1186/s12998-025-00571-6.

## Background

Chiropractic is a widely practiced form of complementary and alternative medicine [1–3], and in 2019 had a global reach of 103,469 chiropractors [4]. Musculoskeletal conditions are the predominant reason for attending chiropractic care, with back pain and neck pain being the most common reason for seeking care [2, 5–9]. Satisfaction with chiropractic care is high and largely perceived as safe and effective [5–8]. Yet, there is still a significant global under-representation of chiropractic in terms of provision of services, education, and legislative and regulatory frameworks [4]. Currently, the chiropractic profession faces recognition and legal challenges in many countries [10], especially in Taiwan [11–13], where musculoskeletal disorders have become an increasing burden [14]. 

The practice of chiropractic is legally recognised in 68 countries, and it is explicitly illegal in 12 countries, including four in Asia (Lebanon, Republic of Korea, Taiwan, and Turkey) [4]. The World Federation of Chiropractic (WFC) classified the legal status of chiropractic care in Taiwan as “unclear and risk of prosecution” [10]. The government has neither recognised chiropractic as a medical profession nor established a licensing pathway for chiropractors. Although patients bear no criminal liability, accredited chiropractors must exercise prudence within the current legal framework to avoid imprisonment for practicing physical therapy without a local license [11]. The Department of Health in Taiwan permits foreign-trained chiropractors to offer “back soothing service” provided they avoid making therapeutic claims or advertising their services as medical treatments [12]. Accordingly, chiropractors are unable to diagnose or treat patients as doctors of chiropractic. Hence, the awareness, acceptance and progress of chiropractic in Taiwan has been slow due to its lack of legal recognition [4, 13]. 

The public may not be against chiropractic but simply have insufficient information to form an opinion [9]. While research on public perception of chiropractic has widely been conducted in the USA, Australia, Canada, New Zealand and The Netherlands [2, 5, 6, 8, 15], there is no information describing the public perception of chiropractic in Taiwan. A Taiwanese-focused investigation is essential to understand the perspectives of the public towards chiropractic, to enable change in the local environment for the profession. Information would serve as a foundation for developing strategies to raise awareness about chiropractic care in Taiwan. Additionally, by gaining a deeper insight into the viewpoints of the Taiwanese population, approaches can be planned by chiropractic stakeholders, such as the WFC, to lobby local authorities on the profession’s validity within the existing healthcare system, and potentially, to its full legalisation. Therefore, the aim of this study is to survey a sample of the Taiwanese population to understand their perceptions of chiropractic in regard to their demographics, utilisation, beliefs and understanding of chiropractic.

## Methods

### Sample and recruitment

The target population were Taiwanese adults worldwide. Sample size was calculated based on the total number of Taiwanese population registered with Taiwan Department of Household Registration in December 2022 (*n* = 23,264,640) [16]. The sample size of 385 was calculated and adequate for the analyses, with a confidence interval of 95% and a 5% margin of error [17]. This study was approved by the CQUniversity Human Ethics Committee (Ref no: 2023-070).

Potential participants were drawn worldwide from a range of Facebook groups predominantly composed of Taiwanese users (Appendix A), through convenience and snowball sampling methods. A recruitment message was posted to outline the study’s purpose of exploring perceptions of chiropractic care among the Taiwanese population, along with eligibility criteria and a link to the online survey. Prior to participation in the survey, a participant information sheet was provided and implied consent was obtained if they chose to participate.

### Design and content of the survey

An anonymous online survey was administered via the cloud-based software platform Qualtrics from January 31 to March 31, 2024. The survey, comprised of 27 close-ended items, was adapted from a previously published survey investigating the public perception of chiropractic in Australia [9]. This survey was tailored to focus on the Taiwanese population and while the survey was translated into Traditional Chinese characters, the official written language in Taiwan (Appendix B), participants were asked to indicate their language preference between English and Traditional Chinese. Five original items, such as nationality and postcode, were removed, while two new items were introduced to identify Taiwanese origin and gender. After adaptation, a pilot test of the survey was conducted with three bilingual Taiwanese individuals, and no further modifications were deemed necessary. The survey took participants approximately 10 to 15 min to complete.

The survey consisted of four multiple choice questions on demographics, including Taiwanese identity, gender, age, and education level. A further 23 single choice questions explored their history of chiropractic use and their understanding about chiropractic care. Seven items assessed participants’ utilisation and perception of chiropractic care. Five questions explored their views of what conditions chiropractors treat and whom they treat, followed by three questions investigating their concerns about chiropractic practice. Five additional items examined participants’ understanding on the education and training required for a qualified chiropractor. The survey concluded with three final items soliciting participants’ opinions on the role for chiropractic in the public healthcare system.

### Statistical analysis

Responses to the survey were transferred to password-protected CQUniversity research data server and deleted from the Qualtrics platform. The collected survey data were then exported into IBM SPSS Statistics 29.0.2.0 for descriptive analysis, including frequencies and cross tabulations. There was no continuous variable in the survey as age data were grouped into brackets, and all categorical variables were reported as number and percentage (n)%. In items where multiple answers were allowed, the total number of responses was taken as the denominator for percentage calculation, instead of using the number of survey participants.

## Results

**Fig. 1:**
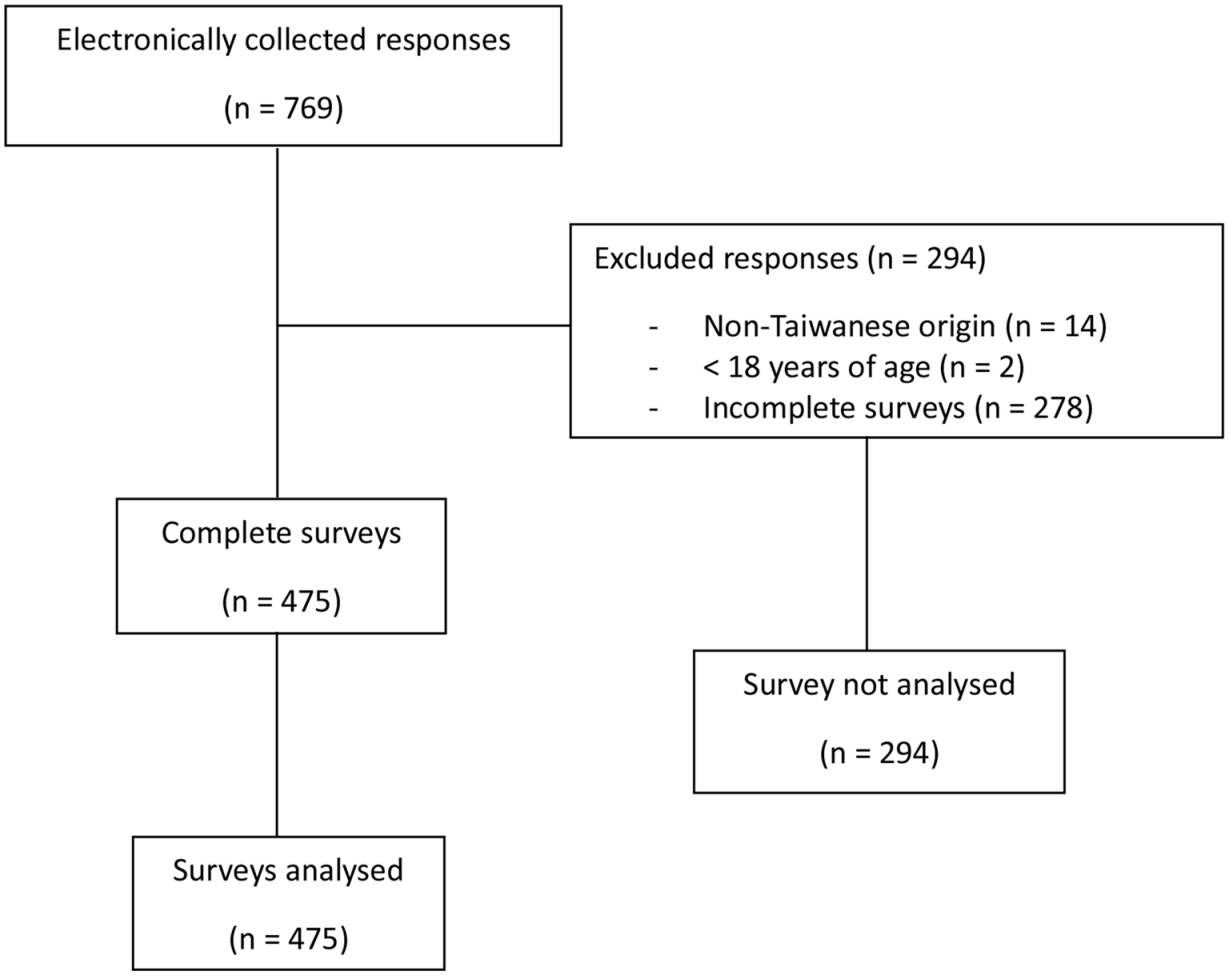
Participation flow diagram

A total of 769 responses were electronically collected. Of the total responses, 294 were excluded due to non-Taiwanese origin (*n* = 14), less than 18 years of age (*n* = 2), and incomplete survey (*n* = 278), resulting in 475/769 (62%) complete responses (Fig. 1). Surveys with all 27 items completed were classified as complete, while any survey with missing responses to the 27 items was classified as incomplete. Among those who did not complete the survey, 21/278 (8%) reported prior experience with chiropractic care, 114/278 (41%) reported no experience, 2/278 (1%) were unsure, and the rest did not respond. According to the IP addresses, 75% of participants (*n* = 355) completed the survey in Taiwan, followed by 16% (*n* = 77) in Australia, and the remaining participants were located in countries across Asia, North America, and Europe (Supplementary Table 1). The majority of participants (95%, *n* = 450) chose to complete the survey in Traditional Chinese, while the remaining 5% (*n* = 25) chose to complete the survey in English. The margin of error for the study was ± 4.5% at a 95% confidence level when the valid sample of 475 was used.

### Demographics

The demographic profile of the 475 participants was 60% female (*n* = 284), 39% male (*n* = 185), and 1% were non-binary or other identifications (*n* = 6). Stratified by age, 50% of the participants were young adults aged 28 to 37 (*n* = 237), followed by 38 to 47 years of age (27%, *n* = 130) (Table 1). In terms of education, 88% (*n* = 419) had either undergraduate or Masters education (Table 1).

**Table 1:** Participant characteristics (*n* = 475)

	*n* (%)
**Age**	
18–27	42 (8.8)
28–37	237 (49.9)
38–47	130 (27.4)
48–57	36 (7.6)
58–67	25 (5.3)
68–77	3 (0.6)
>78	2 (0.4)
**Gender**	
Male	185 (38.9)
Female	284 (59.8)
Non-binary/third gender	3 (0.6)
Prefer not to say	3 (0.6)
**Highest education level**	
Elementary school (equivalent to year 6)	1 (0.2)
Junior high school (equivalent to year 9)	2 (0.4)
Senior high school (equivalent to year 12)	29 (6.1)
University degree (undergraduate)	285 (60)
University degree (Masters)	134 (28.2)
Other	24 (5.1)

### Utilisation and perception of chiropractic

Overall, 62% (*n* = 292) of participants had never visited a chiropractor before, and 55% (*n* = 262) were uncertain or unaware of what chiropractors do. Conversely, 45% (*n* = 213) reported familiarity with chiropractic practices, and 32% (*n* = 151) had utilised chiropractic. The demographic profile of the 151 participants with prior chiropractic use was 54% female (*n* = 81), 44% were aged 28–37 (*n* = 66), and 52% held a university degree (*n* = 79). Among those who had visited a chiropractor, 78% (*n* = 118) expressed satisfaction with the treatment received, 6% (*n* = 9) were dissatisfied, and 16% (*n* = 24) were uncertain. In participants who knew someone who had sought chiropractic care, the care provided was perceived as satisfactory by 60% (*n* = 156), 6% (*n* = 15) expressed dissatisfaction, and 34% (*n* = 88) were not sure. The main reasons for seeking chiropractic care are shown in Table 2, with back pain being the most common reason.

**Table 2:** Reasons for chiropractic care (*n* = 1148)^a^

	*n* (%)
Backache	304 (26.5)
Headache	69 (6)
Neck pain	188 (16.4)
Joint pain	128 (11.1)
Sports injury	198 (17.2)
Muscle pain	141 (12.3)
Other	120 (10.5)

^a^Due to the potential for multiple responses to be selected for this question, the denominator for calculating percentages was based on the total number of responses (*n* = 1148) rather than the number of participants surveyed (*n* = 475)

When suggested to visit a chiropractor, 67% of participants (*n* = 316) expressed interest in learning more about chiropractic care, although 17% (*n* = 82) required persuasion before considering it. Moreover, the remaining participants declined seeing a chiropractor due to reasons such as not having a condition suitable for chiropractic care (8%, *n* = 40), being pain-free (6%, *n* = 27), and feeling physically fit (2%, *n* = 10).

### Perceptions of what and whom chiropractors treat

Participants indicated a belief that chiropractic care addresses the root cause of issues (53%, *n* = 251), rather than solely focusing on symptomatic alleviation (19%, *n* = 90) or preventive measures (15%, *n* = 73). Diverse opinions emerged regarding the frequency of chiropractic visits, with 39% (*n* = 187) considering regular visits necessary, while 30% (*n* = 140) believed visits were only warranted when experiencing pain, and 18% (*n* = 84) remaining uncertain.

Among chiropractic care, soft tissue techniques (24%, *n* = 366), rehabilitation (22%, *n* = 342), and the use of ultrasound or other machines (18%, *n* = 271) were the most frequently used modalities participants perceived chiropractors using (Supplementary Table 2). Notably, 13% (*n* = 204) of participants associated chiropractic care with “bone cracking”. The body parts most commonly reported as associated with chiropractic care were bones (25%, *n* = 367), joints (24%, *n* = 340), the nervous system (23%, *n* = 334), and muscles (19%, *n* = 279) (Supplementary Table 3). Regarding patient case load, self-defined adults (20%, *n* = 427) and middle-aged individuals (20%, *n* = 432) were perceived as the primary groups seeking chiropractic care, followed by elderly (19%, *n* = 392) and adolescents (15%, *n* = 321) (Table 3).

**Table 3:** Perceived patient types treated by chiropractors (*n* = 2124)^a^

	*n* (%)
Elderly	392 (18.5)
Middle aged	432 (20.3)
Adults	427 (20.1)
Adolescents	321 (15.1)
Children	220 (10.4)
Babies	92 (4.3)
Pregnant women	140 (6.6)
People with learning disabilities	100 (4.7)

^a^Due to the potential for multiple responses to be selected for this question, the denominator for calculating percentages was based on the total number of responses (*n* = 2124) rather than the number of participants surveyed (*n* = 475)

### Concerns regarding chiropractic practice

From the total sample, 45% (*n* = 212) of participants considered chiropractic care as safe, 21% (*n* = 101) believed chiropractic care was dangerous, and 34% (*n* = 162) reported they were unsure about its safety. Among those who had visited a chiropractor (*n* = 151), 66% (*n* = 100) considered chiropractic care safe, 16% (*n* = 24) believed it was dangerous, and 18% (*n* = 27) were uncertain. On the other hand, participants who had never experienced chiropractic care (*n* = 292), 36% (*n* = 106) perceived the care as safe, while 25% (*n* = 72) consider it to be dangerous.

The primary concerns of the total sample regarding chiropractic were the cost (36%, *n* = 171), scant knowledge about treatments (27%, *n* = 130), and uncertainty about what treatments can do for them (22%, *n* = 105), whereas 12% (*n* = 56) of participants were without concerns. Opinions of medical doctors would influence 30% (*n* = 144) of participants to stop seeking chiropractic care if they were advised not to seek chiropractic care. Conversely, 32% (*n* = 153) of participants would still choose to visit a chiropractor, while 38% (*n* = 178) were uncertain.

### Perceived education and training of chiropractors

Overall, participants believed a university qualification was required for a qualified chiropractor, indicating either a Masters (58%, *n* = 273) or an undergraduate (40%, *n* = 190) degree. From the total sample 62% (*n* = 294) and 56% (*n* = 268) of participants perceived chiropractors were as well trained as medical doctors and physiotherapists, respectively. On the other hand, 16% (*n* = 77) and 24% (*n* = 112) did not think the training was equal to that of medical doctors and physiotherapists, respectively. In terms of diagnostic competences, 48% (*n* = 229) believed chiropractors could diagnose general health conditions, and 56% (*n* = 268) considered chiropractors competent to perform and interpret X-ray images. Notably, 33% (*n* = 157) and 28% (*n* = 133) of the participants were uncertain about chiropractors’ ability to diagnose general conditions and to take or report X-ray images, respectively.

### Role for chiropractic in the public health system

In total, 80% (*n* = 375) of participants reported that chiropractors should be part of the public health system in Taiwan, 84% (*n* = 400) reported chiropractic care should be covered under Taiwan National Health Insurance, and 92% (*n* = 437) indicated that this would encourage them to use chiropractic care. Moreover, among the 151 of total participants who had previously received chiropractic care, 87% (*n* = 131) endorsed the inclusion of chiropractic in both the healthcare system and Taiwanese National Health Insurance scheme, respectively.

## Discussion

A large proportion of a sample of the Taiwanese population hold a positive view of the chiropractic profession and strongly favour its inclusion in the public healthcare system and Taiwanese National Health Insurance scheme. Participants who had prior chiropractic care demonstrate greater support (87%) which is in line with the findings by Gaumer et al. [18] and Weeks et al. [19] that found previous chiropractic use increases positive narratives. A pressing need to enhance knowledge about chiropractic clinical competency, modalities, and safety among both the public and other healthcare professionals is however needed in Taiwan. Improving public understanding of the profession can lead to more positive perceptions and greater utilisation of chiropractic care [5], while insufficient knowledge poses as a significant barrier for potential patients. [18] Greater awareness of this profession can facilitate access to chiropractic care and help alleviate the musculoskeletal burden on Taiwan’s healthcare system [14]. 

The profile of the sample of Taiwanese participants who had previously sought chiropractic were women, which aligns with global trends where most chiropractic patients are female [2]. Of the 151 participants who had previously sought chiropractic care, they expressed satisfaction with chiropractic care and considered it safe, which is consistent with findings from other countries reported in the literature. For instance, 81% of Australians [9] and 65% of New Zealanders [6] reported satisfaction with chiropractic, and fewer than 10% in the United States [8] and Australia [9] considered chiropractic care dangerous. Our study found that among participants who had seen a chiropractor, satisfaction (78%) notably outweighed dissatisfaction (6%), and over two thirds perceived their treatment as safe, where 16% did not. In contrast, among those who have never seen a chiropractor, one quarter of survey participants perceived chiropractic care as dangerous, and one third thought chiropractic care was safe. Local chiropractic stakeholders in Taiwan may consider organising more marketing and educational events that align with the current legal framework to increase public exposure to chiropractic care and enhance the public’s perceptions of it.

The utilisation of chiropractic care among the sample Taiwanese population is high compared to a global scale. Prior utilisation of chiropractic care was 32% in this study, which is higher compared to Australia (15%) [2],the United States (24%) [20], and globally (22%) [2]. Considering the legal ambiguity surrounding the chiropractic profession in Taiwan since 2003, chiropractors are unable to provide spinal manipulative care legitimately [21]. Instead they are limited to offering alternative physical services as vaguely defined “back soothers” [22]. The lack of recognition for the profession creates a blurred boundary between chiropractic and other forms of non-regulated or traditional manual therapy in Taiwan. For example, a form of traditional manipulative therapy, Tuina, also involves manually repositioning of joints or “bone setting” [23, 24] which resembles chiropractic techniques. Furthermore, censored advertising may also contribute to narrowing the public awareness about chiropractic care, thereby affecting utilisation rates. For instance, most of the participants neither knew nor were certain about what chiropractors do, and even 14% of those who had prior chiropractic care reported uncertainty. These are currently some of the many reasons the Taiwanese public’s utilisation and understanding of chiropractic is hindered. Opportunities exist for global chiropractic stakeholders, such as the WFC, to continue increasing awareness, improving access, and advocating for the regulation of chiropractic care in Taiwan, through initiatives such as online education, campaigns, or hosting international conferences. To alleviate the strain on Taiwan’s healthcare system, Wu et al. [25] emphasise the critical need to bolster the cultivation of a long-term care workforce. According to WHO, qualified chiropractors are equipped with the capabilities to competently perform a differential diagnosis, achieve particular expertise in diagnostic imaging, orthopaedics, pain management and rehabilitation of the neuromusculoskeletal system [26]. Evidently, ready-trained chiropractors in Taiwan can contribute to strengthen the healthcare workforce and improve the wellbeing of the general public, especially in addressing the growing prevalence of musculoskeletal conditions [14]. 

Musculoskeletal conditions were the predominant reason for seeking care from a chiropractor in Taiwan, which complements the global review by Beliveau et al. [2] that also highlighted neck and back-related issues were the most common reason individuals sought chiropractic care worldwide. Interestingly, participants in our study indicated a relatively equal focus that bones (25%), joints (24%), nervous systems (23%), and muscles (19%) are the anatomical parts of the body that chiropractors work on. This pattern suggests that their perceptions of chiropractic management align with the neuromusculoskeletal model defined by the World Health Organization (WHO) [26]. Conversely, in Australia people report that chiropractors work on bones (24%), joints (28%), and muscles (24%), but only 10% knew that chiropractors also addressed components of the nervous system [9]. The increased recognition of the treatment of the nervous system by chiropractors in this Taiwanese sample may result from the advantage of Chinese translation from ‘doctor of chiropractor’ to ‘doctor of spine and nerves’ (脊骨神經醫師) [27]. As 95% of participants answered the survey in Chinese, preference towards the ‘doctor of spine and nerves’ is evidence and may clarify the implicit link between chiropractors and the nervous system in Taiwan.

Divergent reports exist regarding the treatment modalities used by chiropractors in Taiwan, and chiropractors worldwide. The Taiwanese public reported soft tissue massage to be the most frequently used modality by chiropractors, followed by rehabilitation, the use of ultrasound and other machines, and finally chiropractic adjustments. Beliveau et al. [2] reviewed practicing chiropractors in the United States, Canada, Australia, the United Kingdom, and Denmark, and concluded that spinal manipulation is the most used modality, followed by soft-tissue therapy and nutrition or education advice. In Taiwan, the perceptual differences of chiropractic management modalities may result from the prevalent use of folk therapies. Folk medicine practitioners, such as Tuina, meridian massage, and reflexology, also employ soft tissue techniques, rehabilitation, and other complementary tools to provide care [23, 24]. The absence of developed chiropractic education and established regulatory frameworks in Taiwan fails to clearly distinguish the chiropractic profession from other healthcare fields, further weakening the association between chiropractors and manual manipulation. The Ministry of Health and Welfare in Taiwan stated that it was unnecessary to include the chiropractic profession in the healthcare system, as the existing medical workforce was sufficient and capable of providing equivalent care [28]. However, the WHO defines chiropractic as a distinct healthcare profession requiring a minimum of 2,200 hours of training, and considers chiropractic manipulation as therapeutic procedures involving controlled force, leverage, direction, amplitude, and velocity [26]. The WHO-published chiropractic guidelines regulate the acceptable levels of education and competence to ‘facilitate qualified and safe practice of chiropractic as well as to protect the public and patients.’ [26] When patients have inadequate awareness, inappropriate use of the terms ‘chiropractor’ and ‘chiropractic manipulation’ by untrained individuals may lead to patient misconceptions, potentially resulting in harm [29]. A regulatory legal framework is crucial to increase public awareness and ensure standardised manipulative care, and most importantly patient safety [30]. 

The Taiwanese population exhibits a considerable degree of uncertainty regarding their understanding of chiropractic. Almost half of the survey participants, totalling 42%, indicate uncertainty towards the role of chiropractors. In questions pertaining to concerns about chiropractic care, 27% indicated a lack of knowledge about chiropractic, and a third of participants did not know if chiropractors have the ability to diagnose general health conditions. These findings highlight the pronounced necessity for the chiropractic profession in Taiwan to educate the public within the current legal framework. On the other hand, ongoing communication with other healthcare professionals is equally important for raising awareness of the chiropractic profession. More than 65% of participants indicated that they will or may be influenced by the opinions from other medical practitioners when deciding whether to continue chiropractic care. Further, weakened understanding of chiropractic among participants may pose risks to the safety of care users and compromise professional accountability within chiropractic practice.

To the authors knowledge, this is the first survey of Taiwanese adults’ general perceptions of chiropractic. Implications of the findings from this small sample of the Taiwanese public suggests that developing education and marketing campaigns to enhance public awareness of chiropractic and distinguish it from other healthcare professions and/or folk therapy in Taiwan is crucial for increasing recognition of the profession. While these tools and resources will advance the profession, engagement with global stakeholders to increase advocacy is critical. Future research should focus on clarifying the differences between chiropractors and other local healthcare providers and further exploring the potential contributory role of chiropractic within Taiwan’s healthcare system to ensure consistent safe patient care and promote public well-being. Additionally, the perception of other local healthcare providers regarding chiropractic in Taiwan requires investigation, as their opinions influence patients’ decisions to seek chiropractic care.

This study employed convenience and snowball sampling methods via selected Facebook groups, introducing selection bias and limiting generalisability. As a result of recruitment via selected Facebook groups restricting the ability to track participants, the response rate could not be calculated. The sample included Taiwanese worldwide, not just those living in Taiwan, which may further limit the generalisability of the results to the country of Taiwan. The sample size of participants with past chiropractic care experience was small; therefore, the generalisability of their perspectives to the broader population may be significantly limited. Self-reported items within the survey increased recall bias, as participants were required to remember and report past experiences. Survey items were adapted from an English survey and translated into Traditional Chinese not by a professional translator, increasing the risk of misinterpretation. It is also worth noting that this quantitative study exclusively relies on closed-ended questions, thereby leaving more nuanced information unexplored. Lastly, our findings should be interpreted with a degree of caution due to the lack of validity and reliability in the survey instrument. Future studies should focus solely on Taiwan residents and refine surveys rigorously with improved interpretation to yield more meaningful and comprehensive data. Inclusion of open-ended questions and/or qualitative exploration in future surveys would enrich our understanding of public perceptions by allowing for deeper exploration of individuals’ perspectives.

## Conclusion

The overall perception of chiropractic in this Taiwanese sample was positive and demonstrated great acceptance; however, there was a notable lack of understanding regarding the chiropractic profession. Promoting broader awareness of chiropractic clinical competencies, treatment modalities, and safety within the current legal framework should be prioritised to facilitate wider public acceptance. Significantly, clearer recognition may serve as a driving force for obtaining legislative support to integrate the profession in the healthcare workforce. This inclusion would enable a wider range of safe healthcare options to the public and alleviate the burden on the healthcare system.

HC conceived the study, collected the data, performed statistical analyses, and wrote the draft manuscript. KD and MF contributed to the revised manuscript. AQ contributed to study conception and design and revised the manuscript. All authors have read and approved the manuscript and have agreed to be personally accountable for the author’s own contributions.

## Electronic supplementary material

Below is the link to the electronic supplementary material.

Supplementary Material 1

Supplementary Material 2

Supplementary Material 3

Supplementary Material 4

Supplementary Material 5

## Data Availability

No datasets were generated or analysed during the current study.

## Abbreviations

WHO: World Health Organization
WFC: World Federation of Chiropractic
