# Supplementary material for: Public perception of chiropractic in the Taiwanese population: a cross-sectional survey
Source: Chiropr Man Therap. 2025 Mar 11;33:11. doi: 10.1186/s12998-025-00571-6 (PMC11895128; doi:10.1186/s12998-025-00571-6)
Supplement: Supplementary file 1 — Supplementary Material 1 [file 12998_2025_571_MOESM1_ESM.docx]

***Appendix A***

| **Facebook groups with post approval** |
| --- |
| 1. 台灣凱羅健康協會脊骨神經醫學-Chiropractic  (Taiwan Chiropractic Doctors Society) |
| 2. 歐洲的台灣人 (European Taiwanese) |
| 3. 台灣人在布里斯本 (Taiwanese in Brisbane) |
| 4. 台灣人在英國 (Taiwanese in UK) |
| 5. 台灣人在美國 (Taiwanese in USA) |
| 6. 台灣人在巴西網-社團 (Taiwanese Community in Brazil) |
| 7. 台灣人在歐洲互幫社團 (Taiwanese in Europe) |
| 8. 我是台灣人, 我住加拿大 (I'm Taiwanese, and I live in Canada) |
| 9. 台灣人在泰國互助會 (Taiwanese Association in Thailand) |
| 10. 在新加坡工作的台灣人 (Taiwanese work in Singapore) |
| 11. 台灣人在南美洲（拉丁美洲）(Taiwanese in South America) |
| 12. 在台灣的香港人 和 在香港的台灣人  (Hong Kong Residents in Taiwan and Taiwanese in Hong Kong) |
| 13. 小布村愛哈拉 (Taiwanese community in Brisbane, Australia) |
| 14. 台灣同鄉會在墨西哥 Asociacion de los Taiwaneses en México  (Taiwanese Association in Mexico) |
| 15. 紐西蘭台灣之家 (New Zealand Taiwanese Association) |
