# Supplementary material for: Public perception of chiropractic in the Taiwanese population: a cross-sectional survey
Source: Chiropr Man Therap. 2025 Mar 11;33:11. doi: 10.1186/s12998-025-00571-6 (PMC11895128; doi:10.1186/s12998-025-00571-6)
Supplement: Supplementary file 2 — Supplementary Material 2 [file 12998_2025_571_MOESM2_ESM.docx]

***Appendix B***

**Survey: Public perception of chiropractic in the Taiwanese population**

Language preference 語言偏好

[繁體中文/English]

*Skip To: 參與者資訊表If Language preference = 繁體中文*

*Skip To: Participant Information Sheet If Language preference = English*

Q1 Are you a person of Taiwanese origin? 你是台灣人嗎?

[Yes 是/ No 不是]

*Skip To: End of Survey If Are you a person of Taiwanese origin? 你是台灣人嗎? = No 不是*

Q2 Age 年齡

[<18/18-27/28-37/38-47/48-57/58-67/68-77/>78]

Q3 Highest education level 最高學歷

[Elementary school (equivalent to year 6) 小學 /Junior high school (equivalent to year 9) 初中 /Senior high school (equivalent to year 12) 高中 /University degree (undergraduate) 大學 /University degree (Masters) 碩士/ Other 其他]

Q4 Gender 性別

[Male 男 /Female 女/ Non-binary or third gender 非二元性別或第三性別 /Prefer not to say 不願透露]

Q5 Do you know what a chiropractor does? 你知道脊骨神經科醫師是做什麼的嗎？

[Yes 知道 /No 不知道 /Unsure 不確定]

Q6 Have you ever been seen and/or treated by a chiropractor before? 你曾經有過看診和/或接受過脊骨神經科醫師的治療嗎？

[Yes 有 /No 沒有 /Unsure 不確定]

*Skip To: Q8 If Have you ever been seen and/or treated by a chiropractor before? 你曾經有過看診和/或接受過脊骨神經科醫師的治療嗎？ = No 沒有*

Q7 If you have sought chiropractic treatment, why did you seek care? 如果你尋求過脊骨神經科醫師的治療，是出自於什麼原因？

[I knew straight away the chiropractor would help. 我知道脊骨神經科醫師會對我有幫助 /I had heard good things and thought it might help. 我聽過一些好評，並認為這可能會有所幫助 /Last resort as nothing else was working. 因為其他方法都無效，所以這是最後的選擇 /Other 其他]

Q8 Do you know someone who has been treated by a chiropractor before? 你知道身邊有人曾接受過脊骨神經科醫師的治療嗎？

[Yes 有 /No 沒有 /Unsure 不確定]

Q9 What have you, or someone you know, been treated by chiropractors for? (You may tick more than one.) 你自己或你所認識的人，曾經因以下問題接受過脊骨神經科醫師的治療嗎？（你可以勾選多個選項）

[Backache 背痛 /Headache 頭痛 /Neck pain 頸痛 /Other 其他 /Joint pain 關節痛/ Sports injury 運動傷害 /Muscle pain 肌肉疼痛]

Q10 Were you or this person satisfied with the treatment provided by the chiropractor? 你或這個人，對脊骨神經科醫師提供的治療感到滿意嗎？

[Yes 滿意 /No 不滿意 /Unsure 不確定]

Q11 If someone you know suggested you should see a chiropractor, what would your immediate thought be? 如果有人建議你去看脊骨神經科醫師，你的第一個想法會是什麼？

[No, I am fit and healthy. 不，我身體強健 /No, I am not in pain. 不，我沒有疼痛 /I do not have a problem a chiropractor can treat. 我沒有脊骨神經科醫師可以治療的問題 /I would need convincing. 我需要被說服 /I am interested and would want to know more. 我對此感興趣，並且想進一步了解]

Q12 Which of the following do you think chiropractors do? (You may tick more than one.) 你認為以下哪些是脊骨神經科醫師的工作？（你可以勾選多個選項）

[Only “crack bones” (called adjustments) 只是讓關節發出聲響（又稱整脊） /Do massage and other soft tissue techniques 進行按摩和使用其他軟組織放鬆技巧 /Use ultrasound and other machines 使用超聲波和其他儀器 /Use hot and cold packs 使用熱敷和冷敷包 /Rehabilitation 進行康建治療 /Give nutritional advice 提供營養建議 /Unsure 不確定]

Q13 In your opinion, what part of the body do chiropractors work with? (You may tick more than one.) 在你看來，脊骨神經科醫師主要處理身體的哪個部位？（你可以勾選多個選項）

[Bones 骨骼 /Joints 關節 /Muscles 肌肉 /Back only 只有背部 /Nervous system 神經系統 /Arteries, veins and lymphatic system 動脈、靜脈和淋巴系統 /Other 其他]

Q14 Who do you think chiropractors treat? (You may tick more than one.) 你認為脊骨神經科醫師治療的對象是誰？（你可以勾選多個選項）

[Elderly 高齡者 /Middle aged 中年人 /Adults 成年人 /Adolescents 青少年 /Children 兒童 /Babies 嬰兒 /Pregnant women 孕婦 /People with learning disabilities 具有學習障礙的人]

Q15 What do you think chiropractic care does mainly? 你認為脊骨神經科的治療主要是針對什麼？

[Treats symptoms only 只治療病徵 /Treats the cause of the problem 治療病根 /Prevention 預防 /Unsure 不確定]

Q16 How often do you think you would need to see a chiropractor? 你認為你需要多常見脊骨神經科醫師？

[Once every year 每年一次 /A few times a year 每年幾次 /Regularly 定期 /Only when in pain 只在感到疼痛時 /Unsure 不確定 /Never 從不需要]

Q17 Your biggest concern regarding chiropractic work is:  對於脊骨神經科治療，你最大的顧慮是：

[Don’t know anything about it 對該治療不了解 /Unsure what it can do for you 不確定對自己有什麼幫助 /The cost 花費 /I have no concerns 我沒有任何顧慮 /Other 其他]

Q18 From your own understanding of chiropractic, do you think it is dangerous?  就你對脊骨神經科治療的了解，你認為它是危險的嗎？

[Yes 是 /No 不是 /Unsure 不確定]

Q19 If you thought your medical doctor would not like you visiting a chiropractor, would you still see one?  如果你覺得你的主治醫生不想要你去看脊骨神經科醫師，你是否還會去看？

[Yes 會 /No 不會 /Unsure 不確定]

Q20 What education do you think chiropractors need in order to practise?  你認為脊骨神經科醫師需要什麼樣的教育程度才能執業？

[Senior high school (equivalent to year 12) 高中 /University degree (undergraduate) 大學 /University degree (Masters) 碩士]

Q21 Do you think chiropractors are as well trained as medical doctors?  你是否認為脊骨神經科醫師接受的訓練程度與醫學醫生相同？

[Yes 是 /No 否 /Unsure 不確定]

Q22 Do you think chiropractors are as well trained as physiotherapists? 你是否認為脊骨神經科醫師接受的訓練程度與物理治療師相同？

[Yes 是 /No 否 /Unsure 不確定]

Q23 Do you think chiropractors can diagnose general health conditions? 你是否認為脊骨神經科醫師能夠診斷一般的健康問題？

[Yes 是 /No 否 /Unsure 不確定]

Q24 Do you think chiropractors can take x-rays themselves and report on them? 你是否認為脊骨神經科醫師可以自行進行 X 光檢查並報告結果？

[Yes 是 /No 否 /Unsure 不確定]

Q25 Do you think chiropractors should be part of the public health system? 你是否認為脊骨神經科醫師應該成為公共衛生系統的一部分？

[Yes 是 /No 否 /Unsure 不確定]

Q26 Do you think chiropractic should be covered by National Health Insurance in Taiwan? 你是否認為脊骨神經科應該納入台灣的全民健康保險範圍？

[Yes 是 /No 否 /Unsure 不確定]

Q27 If chiropractic were covered by National Health Insurance in Taiwan, would it encourage you to use its services? 如果脊骨神經科被台灣的全民健康保險所涵蓋，這是否會鼓勵你使用其服務？

[Yes 會 /No 不會 /Unsure 不確定]
