# Supplementary material for: Public perception of chiropractic in the Taiwanese population: a cross-sectional survey
Source: Chiropr Man Therap. 2025 Mar 11;33:11. doi: 10.1186/s12998-025-00571-6 (PMC11895128; doi:10.1186/s12998-025-00571-6)
Supplement: Supplementary file 3 — Supplementary Material 3 [file 12998_2025_571_MOESM3_ESM.docx]

| **Supplementary Table 1.** Participant location (n = 475) | |
| --- | --- |
|  | n (%) |
| **Asia** | |
| China | 1 (0.2) |
| Hong Kong | 2 (0.4) |
| Indonesia | 1 (0.2) |
| Japan | 3 (0.6) |
| Malaysia | 3 (0.6) |
| Singapore | 3 (0.6) |
| South Korea | 1 (0.2) |
| Taiwan | 355 (74.7) |
| Thailand | 1 (0.2) |
| Vietnam | 1 (0.2) |
| **Europe** | |
| France | 1 (0.2) |
| Greece | 1 (0.2) |
| Italy | 1 (0.2) |
| Spain | 1 (0.2) |
| United Kingdom | 8 (1.7) |
| **North America** | |
| Canada | 6 (1.3) |
| United States | 7 (1.5) |
| **Oceania** | |
| Australia | 77 (16.2) |
| New Zealand | 2 (0.4) |
