# Supplementary material for: Public perception of chiropractic in the Taiwanese population: a cross-sectional survey
Source: Chiropr Man Therap. 2025 Mar 11;33:11. doi: 10.1186/s12998-025-00571-6 (PMC11895128; doi:10.1186/s12998-025-00571-6)
Supplement: Supplementary file 4 — Supplementary Material 4 [file 12998_2025_571_MOESM4_ESM.docx]

| **Supplementary Table 2.** Perceived modalities used by chiropractors (n = 1529)^a^ | |
| --- | --- |
|  | n (%) |
| Only “crack bones” (called adjustments) | 204 (13.3) |
| Do massage and other soft tissue techniques | 366 (23.9) |
| Use ultrasound and other machines | 271 (17.7) |
| Use hot and cold packs | 167 (10.9) |
| Rehabilitation | 342 (22.4) |
| Give nutritional advice | 132 (8.6) |
| Unsure | 47 (3.1) |
| ^a^ Due to the potential for multiple responses to be selected for this question, the denominator for calculating percentages was based on the total number of responses (n = 1529) rather than the number of participants surveyed (n = 475). | |
