# Supplementary material for: Public perception of chiropractic in the Taiwanese population: a cross-sectional survey
Source: Chiropr Man Therap. 2025 Mar 11;33:11. doi: 10.1186/s12998-025-00571-6 (PMC11895128; doi:10.1186/s12998-025-00571-6)
Supplement: Supplementary file 5 — Supplementary Material 5 [file 12998_2025_571_MOESM5_ESM.docx]

| **Supplementary Table 3.** Perceived body parts treated by chiropractors (n = 1449)^a^ | |
| --- | --- |
|  | n (%) |
| Bones | 367 (25.3) |
| Joints | 340 (23.5) |
| Muscles | 279 (19.3) |
| Back only | 29 (2) |
| Nervous system | 334 (23.1) |
| Arteries, veins, and lymphatic system | 76 (5.2) |
| Other | 24 (1.7) |
| ^a^ Due to the potential for multiple responses to be selected for this question, the denominator for calculating percentages was based on the total number of responses (n = 1449) rather than the number of participants surveyed (n = 475). | |
